# A Single-Layer Multimode Metasurface Antenna with a CPW-Fed Aperture for UWB Communication Applications

**DOI:** 10.3390/mi14020249

**Published:** 2023-01-19

**Authors:** Shu Jiang, Zhiqiang Liu, Huijun Yang, Dongquan Sun

**Affiliations:** 1 School of Information and Communication Engineering, Nanjing Institute of Technology, Nanjing 211167, China; 2 Purple Mountain Laboratories, Nanjing 211111, China; 3 School of Physics, Xidian University, Xi’an 710071, China

**Keywords:** characteristic mode analysis (CMA), coplanar waveguide (CPW), metasurface antenna, ultra-wideband (UWB)

## Abstract

A single-layer multimode metasurface antenna is proposed with a coplanar waveguide (CPW)-fed aperture. The ultra-wideband (UWB) performance is implemented based on a three-step evolution process with the aid of characteristic mode analysis (CMA). Considering the efficient excitation with a fixed feeding structure, the metasurface modal current variation at different frequencies is analyzed and optimized, in addition to that at the resonant frequency. Correspondingly, the metasurface is firstly designed utilizing an array of 4 × 4 patches. Then, the 1 × 3 and the 1 × 1 parasitic patch arrays are located near the edge patches. Finally, every patch is split into two by a center slot along the current distribution of the required polarization. Four resonant modes of the metasurface become more desirable step by step and can be efficiently excited over the entire band. To enhance the impedance matching level, a pair of 5-stage gradient transitions are added to the CPW-fed slot. The slot mode combined with the four modes further improves the bandwidth. The experimental results demonstrate that the proposed antenna exhibits a 3 dB gain bandwidth of over 74% (4.0–8.7 GHz) with a peak gain of 8.2 dBi. The overall dimensions of the prototype are 1.40*λ*_0_ × 1.40*λ*_0_ × 0.075*λ*_0_ (*λ*_0_ is the free-space wavelength at 6 GHz).

## 1. Introduction

Wideband microstrip patch antennas have attracted tremendous attention due to their conformability, compact configuration, low profile, and low cost, along with the development of wireless communication and information technology [1,2]. Novel designs are in great demand for modern multifunctional electronic systems, especially those that cover the ultra-wideband (UWB) positioning channels, Wi-Fi bands (802.11a/ax), and 5G sub-6 GHz bands [3,4,5]. In such designs, it is a great challenge to implement the necessary UWB performance with a simple compact configuration.

For conventional microstrip patch antennas, various techniques have been proposed to deal with the narrow bandwidth drawback, since the antennas excited at the fundamental mode usually suffer from the inherent problem of narrow bandwidth (∼5%) and low gain (∼6 dBi) [6]. Exciting or hybridizing multiple resonant modes has been proposed to increase the operation bandwidth, which mainly includes but is not limited to stacked patches, thick air substrates, reactive slot loading, capacitive coupling feeds, and parasitic resonators [2,7,8,9,10,11]. However, it is still difficult to achieve wide bandwidth (>40%) with low profile (<0.1*λ*_0_) and compactness. The majority of the wideband designs are based involve quality reduction using a thick substrate or a substrate with a low dielectric constant or both.

Metamaterials with unique properties of manipulating electromagnetic waves have provided an effective solution to improving the operation bandwidth [12,13,14]. Several metasurface-based antennas have been proposed for the low-profile and wideband operation [15,16,17,18,19,20,21,22,23,24,25,26,27,28,29,30,31,32,33]. Grid-slotted periodic or quasi-periodic radiating patches inspired by metasurfaces have been demonstrated to achieve a large bandwidth (>30%) and a profile of approximately 0.05*λ*_0_, with two adjacent TM resonant modes (TM10 and antiphase TM20) simultaneously excited [15,16,17,18]. When the sizes of the outer patches are changed to adjust the resonant modes, a bandwidth of approximately 41.3% and 67.3% is available with a profile of 0.054*λ*_0_ and 0.09*λ*_0_, respectively [19,20]. The metasurface also makes it easier to convert the linearly polarized wave into a circularly polarized wave utilizing corner-truncated patches [21,22] or diagonal slots [23,24], and the axial ratio bandwidth of 31.37% is achieved with a profile of 0.038*λ*_0_.

With the aid of the characteristic mode analysis (CMA) [25,26,27,28], the operating mechanisms of the antenna are provided and can guide the optimization of the metasurface [29,30,31,32,33]. In [30], the center patch, the corner patches and the edge patches are accordingly scaled, split and modified, respectively, to optimize the modal current distribution and radiation patterns. The dual bandwidth of 20.7% and 11.3% is obtained with a profile of 0.202*λ*_0_. For the linear-polarized designs utilizing a slot-fed metasurface, the slot mode can also be analyzed with CMA and contribute to the operation band [28,30,31,32,33]. It is also suitable for the dual-polarized antennas utilizing four feeding slots [32,33]. 

However, the above-mentioned analysis focuses on the modal behaviors at the resonant frequencies, which may differ obviously with those over the entire band. Consequently, the desired mode bandwidth is not fully available, which may lead to a narrower antenna bandwidth than the predicted one. In addition, the feeding structure also determines the impedance bandwidth (IBW). It is more difficult to implement UWB impedance matching performance for a single-layer design with an incomplete ground plane.

In this article, a single-layer multimode metasurface with a CPW-fed aperture is proposed for UWB communication applications. It aims at improving the operation bandwidth taking into account the profile, the size, the cross-polarization and the number of the substrates. The theory of characteristic mode is employed for modal analysis. The modal currents at different frequencies, especially at the frequency end, are analyzed and optimized, guiding a three-step evolution process of the metasurface. The design method is verified by a performance comparison among three antennas corresponding to the three-step evolution process. The CPW-fed slot with a pair of 5-stage gradient transitions is proposed for wideband impedance matching. It is etched on the ground to excite the proposed metasurface with multiple modal resonances and boresight radiation. The simulations are carried out by the commercial software Computer Simulation Technology (CST) Microwave Studio and High-Frequency Structure Simulator (HFSS).

## 2. Design of Metasurface Antenna

### 2.1. Antenna Geometry

**Figure 1:**
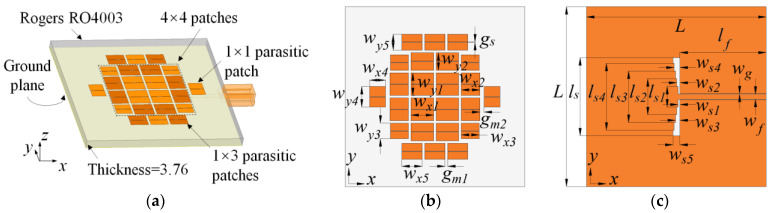
Configuration of the proposed antenna. (**a**) The 3D model. (**b**) Top view with the metasurface (MTS3). (**c**) Bottom view with the CPW-fed slot. *w_x_*_1_ = 8.80, *w_y_*_1_ = 8.80, *w_x_*_2_ = 7.20, *w_y_*_2_ = 6.80, *w_x_*_3_ = 7.00, *w_y_*_3_ = 6.00, *w_x_*_4_ = 6.20, *w_y_*_4_ = 8.00, *w_x_*_5_ = 7.90, *w_y_*_5_ = 6.20, *g_m_*_1_ = 1.00, *g_m_*_2_ = 1.70, *g_s_* = 0.20, *L* = 70.00, *l_s_*_1_ = 8.01, *l_s_*_2_ = 14.01, *l_s_*_3_ = 20.01, *l_s_*_4_ = 26.01, *l_s_* = 30.40, *w_s_*_1_ = 0.80, *w_s_*_2_ = 1.30, *w_s_*_3_ = 1.80, *w_s_*_4_ = 2.30, *w_s_*_5_ = 2.80, *w_g_* = 0.20, *w_f_* = 2.01, and *l_f_* = 33.6, (unit: mm).

The antenna geometry is presented in Figure 1. All designs are arranged on a single-layer substrate of Rogers RO4003 (*ε_r_* = 3.55 and tan *δ* = 0.0027) with a thickness of 3.76 mm. The metasurface and the CPW-fed slot are located on the top and the bottom sides of the substrate, respectively. 

The delicately designed metasurface consists of four parts: (1) an array of 4 × 4 patches in the center with a spacing of *g_m_*_1_, (2) 1 × 3 identical parasitic patches also with a spacing of *g_m_*_1_ on the upper and the lower sides, respectively, (3) 1 × 1 parasitic patches on the left and the right sides, respectively, with a spacing of *g_m_*_2_ to the edge of the 4 × 4 patches, (4) and horizontal split slots with a width of *g_s_*, located in the center of every patch and cutting them into two identical pieces. The metasurface is designed by a three-step evolution process from the original one to the proposed one: (1) the uniform 4 × 4-patch-based metasurface (MTS1), (2) the modified metasurface with parasitic patches (MTS2), and (3) the proposed metasurface with the split slots (MTS3). The modal currents at different frequencies are optimized significantly after the three steps with the aid of CMA.

A CPW-fed slot with a pair of 5-stage gradient transitions is printed in the center of the bottom side. The incident signal propagates in the CPW and couples to the radiation aperture through the bottom slot. The bottom slot introduces a slot mode which also radiates at boresight in the same polarization as the desired four metasurface modes. The detailed analysis is provided in the following section.

### 2.2. Theory of Characteristic Mode

The theory of characteristic mode (TCM) was first initiated in 1965 [34], and then reformulated for computing the CMs for perfect electric conductor (PEC) bodies [25,26]. It has been successfully employed for antenna design, especially for characterizing the truncated metasurface with clear physical insights [28,35]. Furthermore, the CMA is a high-efficiency and feed-less method. The researcher can focus on the pattern configuration at first and then determine the feeding placement accordingly.

According to the TCM, the modal weighting coefficients (MWCs) are employed to characterize the contribution of Mode *n* to the total currents in terms of

(1) $$\alpha_{n}(\omega )=\frac{\int J_{n}(\omega )\cdot E_{i}(\omega )dS}{1+j\lambda_{n}(\omega )}$$

where *λ_n_*(*ω*) is the eigenvalue of Mode *n*. *J_n_*(*ω*) is the *n*th modal current. *E_i_*(*ω*) is the impressed E-field, and *S* is the surface of the conductor area. The amplitude of the first term in the equation is the modal significance (MS) and the second term is the modal excitation coefficient (MEC), which can be defined as

(2) $$\mathrm{MS}=\frac{1}{\left|1+j\lambda_{n}(\omega )\right|}$$

(3) $$V_{i}(\omega )=\int J_{n}(\omega )\cdot E_{i}(\omega )dS$$

The MS characterizes the modal behaviors with no need of any source. When MS = 1, the corresponding mode resonates and radiates with the maximum efficiency. When MS ≈ 0, the corresponding mode barely resonates or radiates. In the antenna design with CMA, a half-power bandwidth is utilized to provide a criterion for evaluating the bandwidth. Each mode within the frequency range is considered significant and in the resonant state when the MS ≥ 0.707. The theoretical mode bandwidth can be described as

(4) $${\mathrm{BW}}_{k}=\left|\frac{f_{H,k}-f_{L,k}}{f_{0,k}}\right|$$

*f_H,k_*, *f_L,k_*, and *f*_0,*k*_ represent the high-frequency end, the low-frequency end, and the center frequency of Mode *k*, respectively. The MS of Mode *k* should be no less than 0.707 in the frequency range from *f_L,k_* to *f_H,k_*. 

For a multimode antenna, every desired resonant mode bandwidth in the target band contributes to the broadband performance. Theoretically, the antenna bandwidth can be implemented when Equation (5) is satisfied.

(5) $$f_{H,p}\geq f_{H},\;f_{L,q}\leq f_{L}$$

Mode *p* and Mode *q* resonates at the highest and the lowest frequencies, respectively, among all the desired modes in the target band. *f_H,p_* and *f_L,q_* represent the high-frequency end in the mode band of Mode *p*, and the low-frequency end in the mode band of Mode *q*, respectively. The MS bandwidth is (*f_H,p_* − *f_L,q_*). The target band is from *f_L_* to *f_H_*. 

However, the implemented bandwidth is usually much lower than the evaluated MS bandwidth (*f_H,p_* − *f_L,q_*). The final limitation of the frequency ends *f_H_* and *f_L_* has not been clearly demonstrated. According to Equation (1), the radiation ability depends not only on the MS, but also on the excitation. When considering a particular feeding structure and location, the modal current *J_n_*(*ω*) is an essential term in determining the excitation efficiency in addition to the MS. It may vary significantly within the MS bandwidth. In many studies, the MS has been paid attention to, while only the modal current at the resonant frequency is given.

In summary, to obtain a broadband performance and avoid gain notches, MWCs should be as high as possible within the MS bandwidth. That is, in the feedless CMA analysis, *J_n_*(*ω*) should be carefully studied and optimized over the whole MS bandwidth accounting for the same assumed feeding structure with that of the resonant frequency.

### 2.3. CMA of Uniform 4 × 4-Patch-Based Metasurface (MTS1)

**Figure 2:**
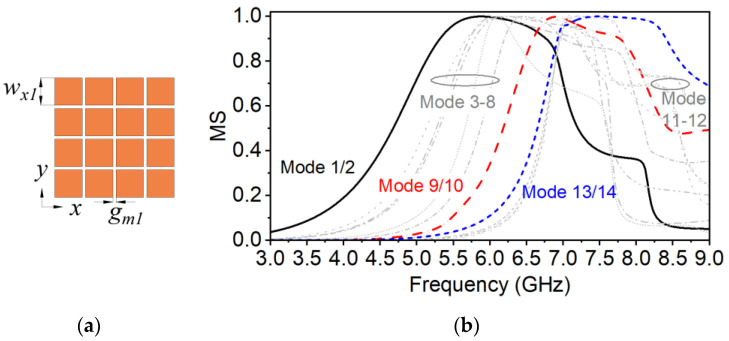
Configuration and the MSs of the uniform 4 × 4-patch-based metasurface (MTS1). (**a**) Metasurface topology; (**b**) the MS.

The proposed antenna can be viewed as a type of grid-slotted patch [36]. According to the TCM, the bandwidth is broadened along with the increasing of the unit number in the patch array. When the unit number increases from 1 × 1 to 4 × 4, the mode bandwidth can be broadened significantly. When it continues to increase up to 6 × 6, the current intensity on the outer patches is relatively weak and the further improvement is small in the bandwidth. Therefore, a uniform 4 × 4-patch-based metasurface (MTS1) design is utilized in the first step for the required bandwidth and compact size. To illuminate the three-step evolution process of the proposed antenna, MTS1 is analyzed at first without the feeding structure as shown in Figure 2. 

**Figure 3:**
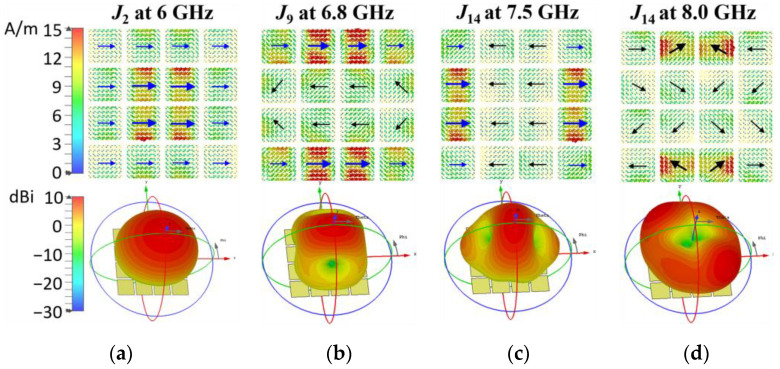
Modal currents *J_n_*(*ω*) and radiation patterns of MTS1 at the resonant frequencies and the frequency end. (**a**) Mode 2 at 6 GHz, (**b**) Mode 9 at 6.8 GHz, (**c**) Mode 14 at 7.5 GHz, and (**d**) Mode 14 at 8.0 GHz.

MTS1 in Figure 2a contains an array of 4 × 4 squared patches with dimensions of *w_x_*_1_×*w_x_*_1_. The edge-to-edge spacing between the adjacent patches is *g_m_*_1_. The MSs of the first 14 modes, as shown in Figure 2b, are calculated and sorted at 6 GHz by the multilayer solver in CST, where the ground plane and dielectric layers are infinitely extended in x- and y-directions. The Modes 1/2, 9/10 and 13/14 are pairs of degenerate modes, respectively. The fundamental Modes 1/2 are designed to be resonant at approximately 6 GHz by changing the size of the patches. Assuming that only the polarization along the x-axis is desired, the Modes 2, 9 and 14 are investigated, while the other modes are not taken into account according to Equation (3). The corresponding modal currents and radiation patterns of the investigated modes at resonant frequencies are plotted in Figure 3a–c. According to Equation (4), the mode band of Modes 2, 9 and 14 are approximately 4.9–7.0 GHz, 6.4–8.0 GHz, and 6.8–8.8 GHz, respectively. Mode 2 has a good main lobe at the resonant frequency 6 GHz in Figure 3a, and its modal current *J*_2_ remains in phase along the x-axis over 4.9–7.0 GHz. Modes 9 and 14 in Figure 3b,c have split main lobes due to the inversed current distribution, which may affect the total radiation pattern. In addition, the modal current *J*_14_ vary obviously along with the frequency increase. When the frequency increases up to 8.0 GHz, the inversed current intensity is enhanced. The distribution of the current phase and intensity becomes symmetric to the center. Then, the boresight gain is nearly 0 according to the radiation pattern as shown in Figure 3d. Consequently, the realized gain of the antenna may decrease significantly beyond the mode band of Mode 2 and the available antenna band cannot cover up to 8.0 GHz.

In this condition, the next step is to optimize the modal currents especially those around the upper frequency end and reduce the inversed current components.

### 2.4. CMA of Modified Metasurface with Parasitic Patches (MTS2)

**Figure 4:**
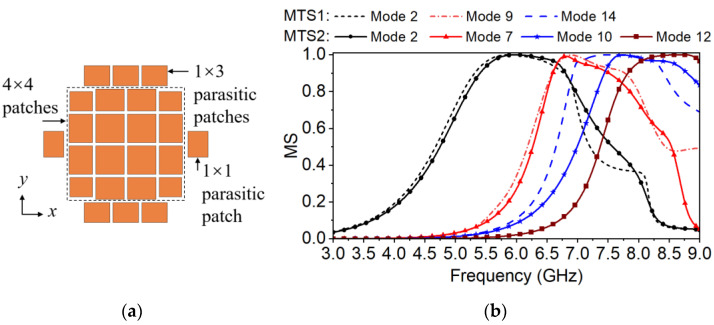
Configuration and the MSs of the modified metasurface with parasitic patches (MTS2). (**a**) Metasurface topology of MTS2. (**b**) The MSs of MTS1 and MTS2.

The modified metasurface with parasitic patches (MTS2) is shown in Figure 4a. In comparison with MTS1, the corner and the edge units of the 4 × 4 patches are miniaturized. The resonant frequencies and modal patterns can be changed by varying the size of the patches [30]. The 1 × 3 parasitic patches are added to the upper and the lower sides. The 1 × 1 parasitic patches are added to the left and the right sides. Coplanar parasitic patches loaded around the metasurface units can help to introduce extra in-band resonances for wide bandwidth and simultaneously generate upper-edge gain nulls [37]. The 1 × 3 and 1 × 1 parasitic patches are adjusted to improve the bandwidth while move the generated gain nulls out of the operation band. The dimensions of MTS2 are the same as those of the proposed metasurface in Figure 1.

**Figure 5:**
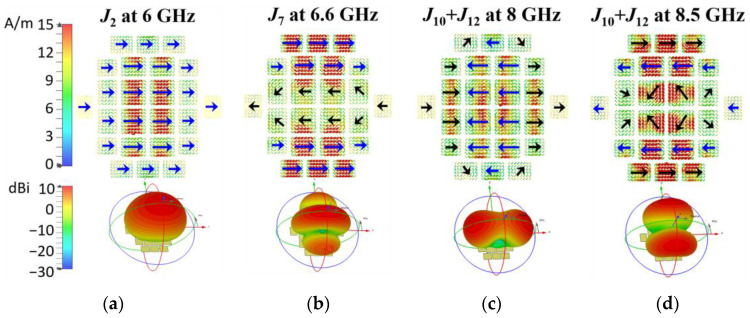
Modal currents *J_n_*(*ω*) and radiation patterns of MTS2 at the resonant frequencies and at the upper frequency ends (8.0 and 8.5 GHz). (**a**) Mode 2 at 6.0 GHz, (**b**) Mode 7 at 6.6 GHz, (**c**) Mode 10 + 12 at 8.0 GHz, and (**d**) Mode 10 + 12 at 8.5 GHz.

The MSs of MTS2 are calculated and the selected four modes—Modes 2, 7, 10, 12—are shown in Figure 4b. They might be well excited by the assumed feeding slot. The corresponding modal currents and the radiation patterns are depicted in Figure 5. It is obvious that Modes 2, 7, and 10 of MTS2 correspond to Modes 2, 9, and 14 of MTS1, respectively, while Mode 12 is new added. The fundamental Mode 2 resonating at approximately 6 GHz still remains the desired modal current and radiation pattern. It remains in phase along the x-axis over the mode bandwidth. Mode 7 resonates at approximately 6.6 GHz has distinguished side lobes in the H plane, which need to be suppressed in the next step. Mode 10 and Mode 12 resonated at approximately 7.5 and 8.0 GHz, respectively. Their mode currents vary significantly in the mode band especially when the frequency increases to the upper frequency end. The mode behavior of MTS2 over 7.5 GHz is mostly determined by both Modes 10 and 12 with the MS close to 1. Therefore, their mode currents and radiation patterns are combined for analysis and illumination, as shown in Figure 5c,d. *J*_10_ + *J*_12_ is acceptable until the frequency increases over 8.0 GHz. It is obvious that *J*_10_ + *J*_12_ is enhanced along the x-axis on most patches compared to *J*_14_ in Figure 3, and can be efficiently excited by the assumed feeding slot. However, the inversed currents are almost as strong as the required ones. Although the main lobe is not split at 8.0 GHz, the boresight gain is relatively low. The radiation pattern is barely acceptable. At 8.5 GHz, the beam is split into two with boresight gain of nearly 0, which is not acceptable.

In general, compared to MTS1, the effective bandwidth of MTS2 is improved by optimizing the modal current distribution. However, the boresight gain at 8.0 GHz is relatively low and the operation band cannot cover to 8.5 GHz. It is necessary to reduce the inversed modal currents in the next step.

### 2.5. CMA of Proposed Metasurface with Split Slots (MTS3)

**Figure 6:**
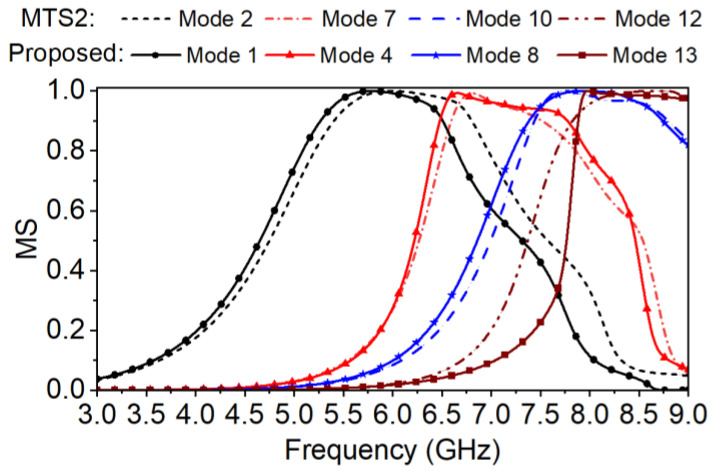
The MSs of the proposed metasurface (MTS3) in comparison with those of MTS2.

**Figure 7:**
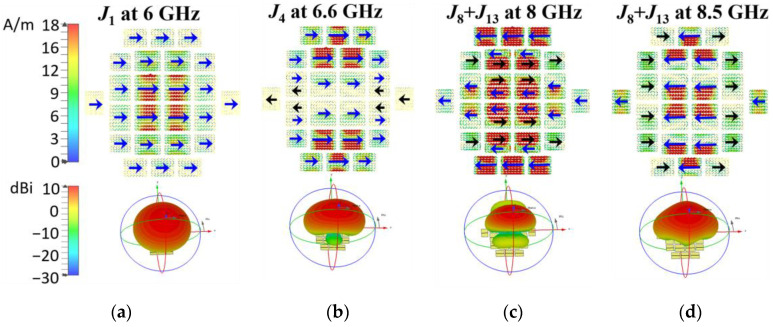
Modal currents *J_n_*(*ω*) and radiation patterns of the proposed metasurface (MTS3) at the resonant frequencies and at the upper frequency ends (8.0 and 8.5 GHz). (**a**) Mode 1 at 6 GHz, (**b**) Mode 4 at 6.6 GHz, (**c**) Mode 8 + 13 at 8.0 GHz, and (**d**) Mode 8 + 13 at 8.5 GHz.

The metasurface of the proposed antenna is shown in Figure 1. To weaken the modal currents along the y-axis or inversed while not to decrease the wanted ones, every patch of the metasurface is cut into two halves in the center by a split slot *g_s_* along the x-axis [20,30]. The MSs of the selected Modes 1, 4, 8 and 13 are calculated as shown in Figure 6. The corresponding modal currents and the radiation patterns are depicted in Figure 7.

In Figure 6, the MSs of the fundamental Mode 1 and the higher Modes 4 and 8 are still similar to those of the Modes 2, 7, and 10 of MTS2, and also resonant at approximately 6 GHz, 6.6, and 7.5 GHz, respectively. Mode 13 is moved to the upper frequency band compared to Mode 12 of MTS2.

By comparing the modal currents of MTS2 in Figure 5 and those of the proposed metasurface in Figure 7, it is obvious that the modal current *J*_1_ of the proposed metasurface on every patch still remains distributed in the same direction, forming required radiation pattern. It still barely changes within the mode bandwidth. According to *J*_4_ and *J*_8_+*J*_13_ in Figure 7, the modal current components along the y-axis is eliminated and the inversed ones are reduced. Therefore, the side lobe level is decreased while the boresight gain is enhanced. The cross-polarization level can also be improved.

In general, for the proposed metasurface, both the wide MS bandwidth and the available modal currents *J_n_*(*ω*) ensure that the effective antenna bandwidth can cover to 8.5 GHz.

### 2.6. Design of the CPW-Fed Slot

Various transmission lines have been employed for wave propagation with acceptable IBW and loss, including the microstrip, the strip lines, the coaxial probe, the CPW, the substrate-integrated waveguide (SIW), and the substrate-integrated gap waveguide (GW) [19,29,30,31,35,38]. According to the concept of the hybrid and the multimode antennas, the transition to a feeding slot is mostly required for driving the metasurface. The bandwidth can be extended to further cover the lower frequency band, since another slot mode is accordingly introduced. It also radiates at boresight in the same polarization as the excited metasurface modes. The slot-coupled antennas are simple and compact for the single-layer and linear-polarized applications. 

In this design, a slot is etched on the bottom ground plane along the y-axis, underneath the middle gap of the metasurface. The CPW is utilized for signal propagation, which is practical for the single-layer substrate. The slot modal behavior analysis and the impedance matching are two essential issues. 

**Figure 8:**
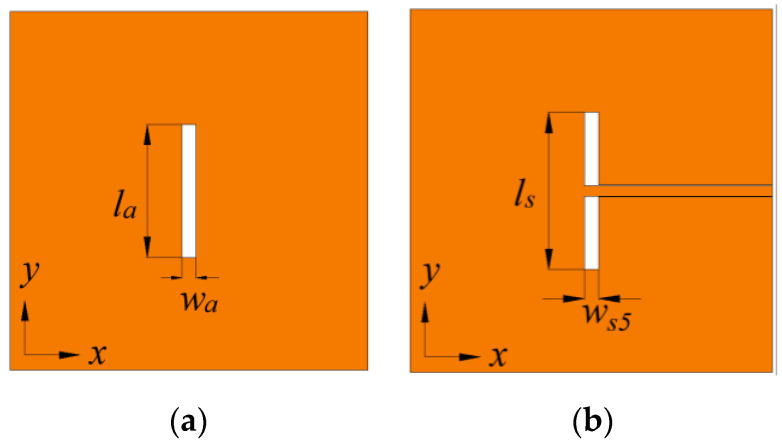
Design steps of the CPW-fed slot on the ground plane. (**a**) Single slot without CPW. (**b**) Original CPW-fed slot without the 5-stage gradient transitions. *l_s_*_1_ = 28.00, *w_a_* = 2.80, (unit: mm).

**Figure 9:**
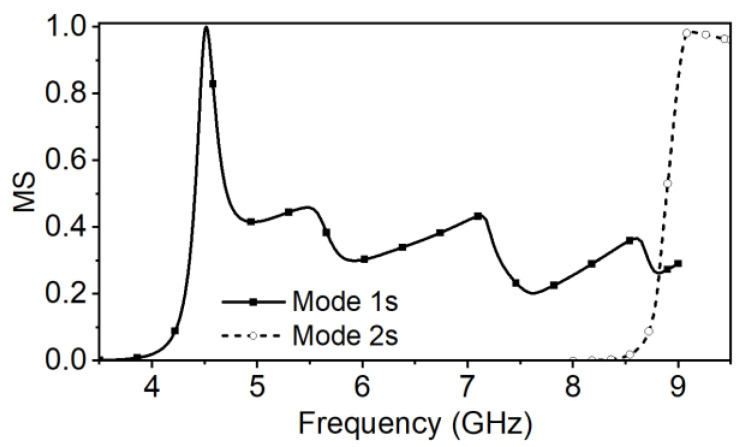
The MSs of the half-wavelength mode (Mode 1s) and the full-wavelength mode (Mode 2s) of the single slot.

There are three steps in design of the CPW-fed slot. Firstly, the single slot without CPW as shown in Figure 8a is analyzed with CMA. The lower frequency end is limited by the half-wavelength mode of the slot. The length *l_a_* must be greater than half a wavelength so that the virtual open-circuit condition at both sides can be satisfied to ensure efficient coupling of energy from the transmission line to the feeding slot. In addition, to ensure in-phase magnetic current along the feeding slot thus the in-phase electric current over the metasurface in the operation band, the full-wavelength mode of the slot must be higher than the upper frequency end [28]. The MSs of the half-wavelength and the full-wavelength modes, denoted as Mode 1s and Mode 2s, respectively, are shown in Figure 9. The resonant frequencies are located at approximately 4.5 and 9.1 GHz, respectively. Mode 1s can excite a mode that has a broadside radiation pattern. When combined with the proposed metasurface, the operation band can be no less than 4.5–8.5 GHz. The realized gain may decrease beyond the range.

**Figure 10:**
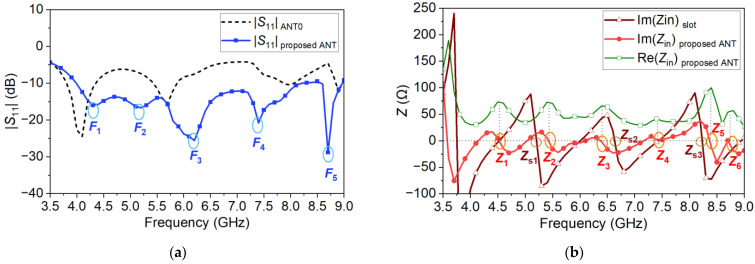
(**a**) Simulated |*S*_11_| of ANT0 and the proposed antenna. (**b**) Simulated complex impedance of the proposed antenna and the proposed CPW-fed slot.

Secondly, the original CPW-fed slot in Figure 8b is utilized to slightly adjust the slot size, simulated with the proposed metasurface. The impedance bandwidth reaches 3.5–9.0 GHz when the return loss is compromised to approximately 5 dB, marked by ANT0 in Figure 10a. Finally, accounting for the structural discontinuity at the shorted end of the CPW, a pair of 5-stage gradient transitions are added as shown in Figure 1c. The design offers additional design flexibility for impedance matching. The return loss of the proposed antenna is significantly improved, better than 10 dB over 4.0–9.0 GHz as depicted in Figure 10a. It exhibits five resonant frequencies at 4.3, 5.2, 6.2, 7.4 and 8.7 GHz, marked by *F*1–*F*5. 

To further demonstrate the operating mechanism of the proposed antenna, the complex input impedance (*Z_in_* = *R_in_* + *jX_in_*) of the proposed CPW-fed slot without metasurface and for the proposed antenna is shown in Figure 10b. At the resonant frequencies of an antenna, one may obtain *X_in_* = 0 and negative-slope dispersion [16,39]. Within the frequency band with |*S*_11_| = −10 dB, we can find three resonant modes of the CPW-fed slot (marked by *Z_s_*_1_–*Z_s_*_3_), and six resonant modes of the proposed antenna (marked by *Z*_1_–*Z*_6_) with *X_in_* = 0 and negative slopes. The frequencies of the resonant modes *Z*_2_, *Z*_3_ and *Z*_5_ are close to those of *Z_s_*_1_, *Z_s_*_2_ and *Z_s_*_3_, respectively. They are mainly or partially generated by the slot mode. In addition, the resonant mode located at 3.55 GHz is also generated by the slot mode while it is out of the operation band. The frequencies of *Z*_1_–*Z*_4_ are almost the same with the resonant frequencies *F*_1_–*F*_4_, respectively. *F*_1_–*F*_4_ work at the resonant modes *Z*_1_–*Z*_4_, respectively. *F*_5_ is close to the frequencies of *Z*_5_ and *Z*_6_. It works at the combination of the resonant mode *Z*_5_ and *Z*_6_. 

**Figure 11:**
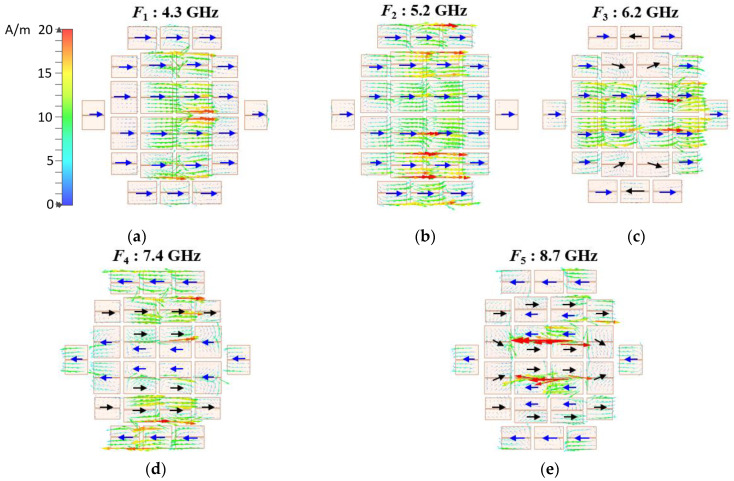
Simulated surface currents of the proposed antenna at resonant frequencies. (**a**) *F*_1_: 4.3 GHz, (**b**) *F*_2_: 5.2 GHz, (**c**) *F*_3_: 6.2 GHz, (**d**) *F*_4_: 7.4 GHz and (**e**) *F*_5_: 8.7 GHz.

The currents of the proposed antenna at *F*_1_–*F*_5_ are investigated and shown in Figure 11 to distinguish the effect of the slot and the metasurface modes. The current distribution at *F*_1_ and *F*_4_ are the same as the modal current *J*_1_ in Figure 7a and *J*_8_+*J*_13_ in Figure 7c, respectively. The current at *F*_2_ on the center patches is weakened, same as the modal current *J*_4_ in Figure 7b, while it turns to be in phase on the entire metasurface. It is generated by mixing the slot mode and the metasurface Mode 4. The current at *F*_3_, relatively strong on the center patches, is generated by the slot mode. It is in phase except for that on the upper and the bottom edge patches marked by black arrows in Figure 11c. The inversed current is caused by the interaction between the narrow side of the bottom slot and the metasurface. The current at *F*_5_ is similar to the modal current *J*_8_+*J*_13_ in Figure 7c, while it is relatively strong on the center patches. It is generated by mixing the slot mode and Modes 8 and 13 of the metasurface. The wide bandwidth is achieved by combining the four modes of the metasurface and the modes of the slot. These results demonstrate the validity of the above analysis about the operating mechanism of the proposed metasurface antenna.

## 3. Simulations and Experiments

**Figure 12:**
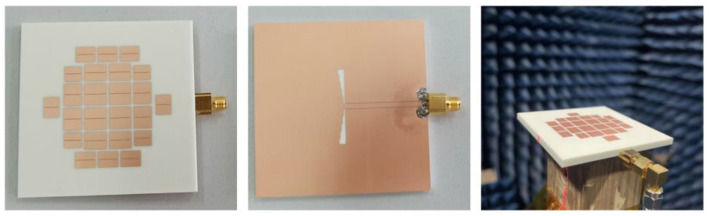
Photograph of the proposed antenna.

**Figure 13:**
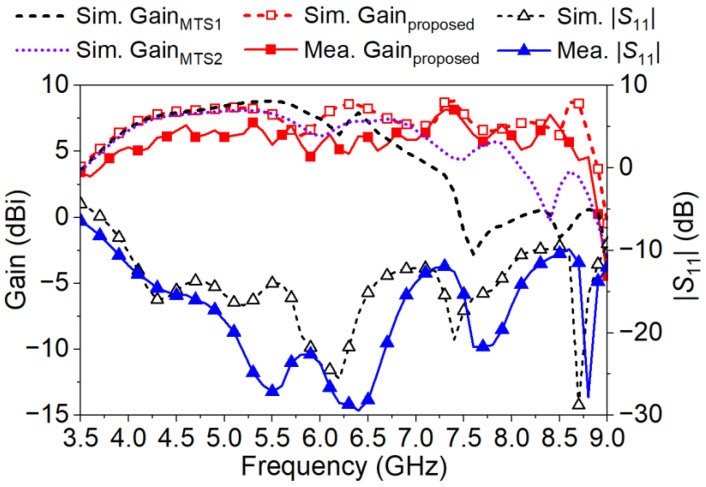
Measured and simulated boresight gains, |*S*_11_| of the proposed antenna. Simulated boresight gains of antennas with MTS1 and MTS2 are also shown.

A prototype of the proposed metasurface antenna was fabricated and measured as presented in Figure 12. The coaxial cable with a characteristic impedance of 50 Ω is used to feed the antenna in the application. The simulated and measured return loss, realized gain are all depicted in Figure 13. The simulated gains of MTS1 and MTS2 with the proposed CPW-fed slot are also illustrated in Figure 13. By comparing the realized gain, it is obvious that antennas utilizing MTS1 or MTS2 cannot be efficiently excited over 8 and 8.5 GHz, respectively. In contrast, the boresight gain of the proposed antenna is flat over 4.5–8.5 GHz and then decreases beyond the range. The results are in consistent with the analysis in Section 2. 

**Figure 14:**
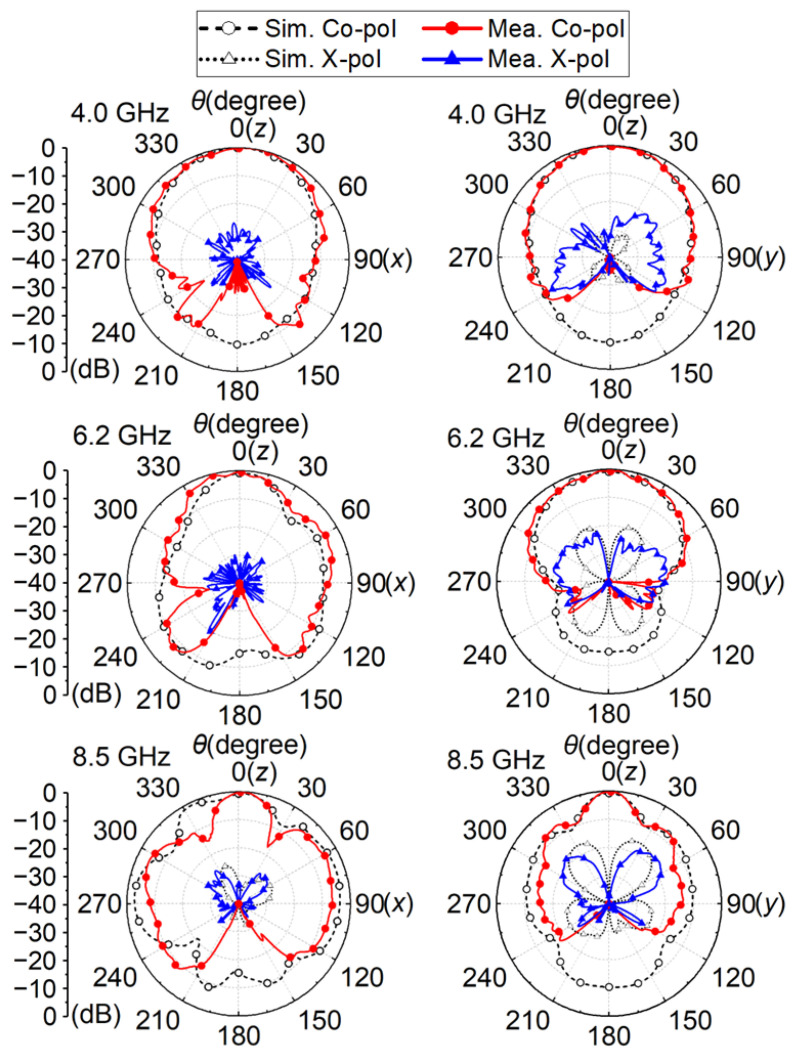
Simulated and measured E-/H- plane (xoz/yoz -plane) cross- and co-polarization radiation patterns of the proposed antenna.

In Figure 13, the measured IBW of the proposed antenna is 3.9–9.0 GHz (79%) with |*S*_11_| of −10 dB. The measured 3 dB gain bandwidth is 4.0–8.7 GHz (74%) with the peak gain of 8.2 dBi. The normalized radiating patterns of the proposed antenna on the E- and H-planes at 4.0, 6.2, and 8.5 GHz are plot in Figure 14. The measured co-polarization is in good agreement with the simulated one within the ±120° beam range. The back-lobe level is approximately 20 dB lower than the simulation, because the turntable and the absorbers on the backside of the antenna weakened the backward radiation. Although the radiation on the E-plane is not ideally symmetric due to the CPW and the connector on one side of the substrate, the maximum radiation is in the desired boresight direction across the operating band. The measured cross-polarization is better than −30 dB at boresight over the operating band. It reaches −30 dB (−20 dB) on the E-plane (H-plane) within the ±30° beam range. The difference between the simulations and the measurements is acceptable. It is caused by the manufacturing tolerance, the fluctuations in the substrate permittivity, the metal loss and the measurement errors.

Table 1 demonstrates the performance of some reported works on relative microstrip antennas. In comparison with the other antennas with a single-layer or dual-layer substrate, it is obvious that the proposed antenna achieves a significant improvement on the wide bandwidth and the low profile. Compared with the antennas with a single-layer substrate [16,18,19,20], the total dimensions and the peak gain of the proposed antenna are comparable while the bandwidth is much broader.

## 4. Conclusions

A single-layer multimode metasurface antenna with a CPW-fed slot has been proposed, analyzed and measured for high performance and low profile. The UWB performance is implemented based on the three-step evolution process in the metasurface design, and the impedance matching in the CPW-fed slot design, and the multimode combination. The three-step evolution process is guided by the CMA-based optimization. The modal current at different frequencies, in addition to the resonant frequency, is taken into account considering efficient excitation with a fixed feeding structure. In design of the CPW-fed slot, a pair of 5-stage gradient steps are added to improve the impedance matching level. The slot mode combined with the four metasurface modes further improves the operation bandwidth. The proposed antenna achieves a wide impedance bandwidth above 79% and a wide 3 dB realized gain bandwidth of 74% with low profile of 0.075*λ*_0_. The proposed antenna features the advantages of wide band, low profile, low cost and easy fabrication. It provides a competitive solution to UWB communication applications.

## Figures and Tables

**Table 1:** Comparison of merits with some previously reported metasurface antennas.

Ref	Total Size (*λ*_0_ × *λ*_0_ × *λ*_0_)	IBW	Peak Gain (dBi)	XP (dB)	NO. of Substrate Layer
[16]	1.10 × 1.10 × 0.052	36.5% (3.96–5.73 GHz)	10.45	−15	1
[17]	1.63 × 1.63 × 0.070	28% (4.41–5.85 GHz)	12.1	<−30	2
[18]	1.00 × 1.10 × 0.058	26.5% (5.0–6.6 GHz)	8.68	−20	1
[19]	1.28 × 1.28 × 0.090	67.3% (4.81–9.69 GHz)	9.18	−15	1
[20]	1.40 × 1.22 × 0.054	41.13% (4.48–6.80 GHz)	10.14	−17	1
[28]	1.78 × 1.78 × 0.070	31% (4.75–6.50 GHz)	14.5	−30	2
[38]	1.29 × 1.01 × 0.044	57.3% (2.94–5.30 GHz)	>5.26	−18	2 + air gap
This work	1.40 × 1.40 × 0.075	79% (3.9–9.0 GHz)	8.2	−20	1

*λ*_0_ is the free-space wavelength at the center frequency.

## Data Availability

Not applicable.
